# Preliminary Exploration of the Cause of Liver Disorders During Early Stages in COVID-19 Patients

**DOI:** 10.3389/fmed.2020.00501

**Published:** 2020-08-07

**Authors:** Yuan Gao, Qi Li, Hongbo Shi, Yingmei Feng, Tong Zhang, Yu Chen, Lianchun Liang, Dexi Chen, Hao Wu, Ronghua Jin, Xiaojie Huang

**Affiliations:** ^1^Difficult & Complicated Liver Diseases and Artificial Liver Center, Beijing Youan Hospital, Capital Medical University, Beijing, China; ^2^Department of Hepatology and Gastroenterology, Beijing Youan Hospital, Capital Medical University, Beijing, China; ^3^Beijing Youan Hospital, Beijing Institute of Hepatology, Capital Medical University, Beijing, China; ^4^Center for Clinical Research, Beijing Youan Hospital, Capital Medical University, Beijing, China; ^5^Center for Infectious Diseases, Beijing Youan Hospital, Capital Medical University, Beijing, China; ^6^Department of Infectious Disease, Beijing Youan Hospital, Capital Medical University, Beijing, China

**Keywords:** COVID-19, immune response, liver injury, early stage, cytokines

## Abstract

**Background:** Abnormal liver function is a common indication of coronavirus disease 2019 (COVID-19) patients. Two proposed mechanisms are liver injury mediated by angiotensin-converting enzyme 2 (ACE2) and the involvement of the systemic immune response. We investigated the role played by these to determine the cause of liver abnormality in the early stages of COVID-19.

**Methods:** A cross-sectional study was conducted among confirmed cases of COVID-19 at Beijing Youan Hospital from January 21, 2020, to February 24, 2020. We compared clinical characteristics, viremia status, and cytokine profile on admission between patients with and without liver disorder.

**Results:** Of the 44 COVID-19 patients analyzed, there were no differences in the clinical symptoms and signs, disease severity, or computed tomography (CT) image features between the two groups. Lymphopenia was more common in the liver disorder group. Further, C-reactive protein levels were much higher in the hepatic disorder group, with significantly higher concentrations of IL-6, IL-10, and M-CSF. Viremia was detected in only 7% of patients.

**Conclusions:** Due to the infrequency of viremia, ACE2-mediated viral hepatitis does not seem to account for the commonly observed liver disorders in COVID-19 patients. By contrast, a dysregulated immune response may be a crucial pathogenic factor for liver disorder in the early stages of COVID-19.

## Introduction

In the largest-scale study to date of the clinical characteristics of coronavirus disease 2019 (COVID-19) patients in China, about one-fifth of cases featured elevated alanine aminotransferase (ALT) levels on admission, with a higher prevalence in critically ill patients (1). The mechanism underlying this abnormal liver function remains a matter of debate. Because angiotensin-converting enzyme 2 (ACE2) has been confirmed to be the receptor for the entry of severe acute respiratory syndrome coronavirus 2 (SARS-CoV-2) into the cells (2) and is expressed in multiple organs and tissues (3), such as cholangiocytes, which are involved in many aspects of liver physiology, including regeneration and adaptive immune response mechanisms, and dysfunction of cholangiocytes can cause hepatocytes damage, it has been proposed that the virus could cause direct extrapulmonary-organ dysfunction, such as liver injury. But it is important to note that an elevation of cholestatic markers is not found among all COVID-19 patients with liver disorders (4). It is also possible that hepatic damage relates to the systemic immune response (5, 6). After SARS-CoV-2 invades airway epithelial cells, partial patients show aggressive inflammatory responses. The vast release of cytokines by the immune system in response to the viral infection could result in a cytokine storm, and compromise multiple organs, including the liver (7). To clarify which factor plays the most crucial role in liver disorders during the early stages of COVID-19 and provide scientific evidence to aid physicians' decision-making in clinical practice, we conducted a cross-sectional study to compare viremia status and cytokine levels between COVID-19 patients with and without liver disorder on admission.

## Patients and Methods

From January 21, 2020, to February 24, 2020, a total of 92 patients with COVID-19, confirmed by real-time RT-PCR tests of nasopharyngeal swabs, were admitted to Beijing Youan Hospital in China. Written informed consent was obtained from 44 patients who were enrolled in the study, among whom 22 had liver disorder, defined as abnormal plasma levels of ALT exceeding 40 U/L in women or 50 U/L in men (Siemens advia2400, Germany), regardless of AST level.

Epidemiological, demographic, clinical, laboratory, and radiological data were extracted from the patients' electronic medical records.

Plasma samples drawn from all subjects at admission were stored in the biobank of Beijing Youan Hospital. The levels of the cytokines and chemokines IL-1α, IL-1β, IL-1RA, IL-3, IL-5, IL-6, IL-7, IL-8 (also known as CXCL8), IL-9, IL-10, IL-12p40, IL-12p70, IL-13, IL-15, IL-17A, eotaxin (also known as CCL11), FGF2, G-CSF (CSF3), M-CSF, IFN-α2, MCP-1 (CCL2), MIP-1α (CCL3), MIP-1β (CCL4), TNF-α, TNF-β, and VEGF-A in plasma taken on admission were analyzed using the Luminex 200TM assay (Millipore, Billerica, USA), according to the manufacturer's instructions.

A 200 μL plasma sample from each patient was analyzed using a Duplex Real-time PCR Diagnostic Kit for Rapid Detection of 2019-nCoV ORF1ab/N gene (A7712RC-50T, XABT, Beijing, China). Viremia was defined as a positive result for real-time RT-PCR in the plasma sample.

### Study Approval

The study protocol was approved by the Ethics Committee for Beijing Youan Hospital, Capital Medical University.

### Statistical Analyses

Continuous variables are expressed as medians (IQRs) and were compared using the Mann–Whitney *U*-test. Categorical variables are expressed as numbers (percentages) and were compared using the χ^2^ test or Fisher's exact test. All comparisons were between patients with and without liver disorders. Boxplots are given to describe the concentrations of cytokines and chemokines in plasma. Two-sided α <0.05 were considered to indicate statistical significance. Statistical analyses were performed using IBM SPSS Statistics software version 21.0 and Graphpad Prism version 8.0.2.

## Results

**Figure 1:**
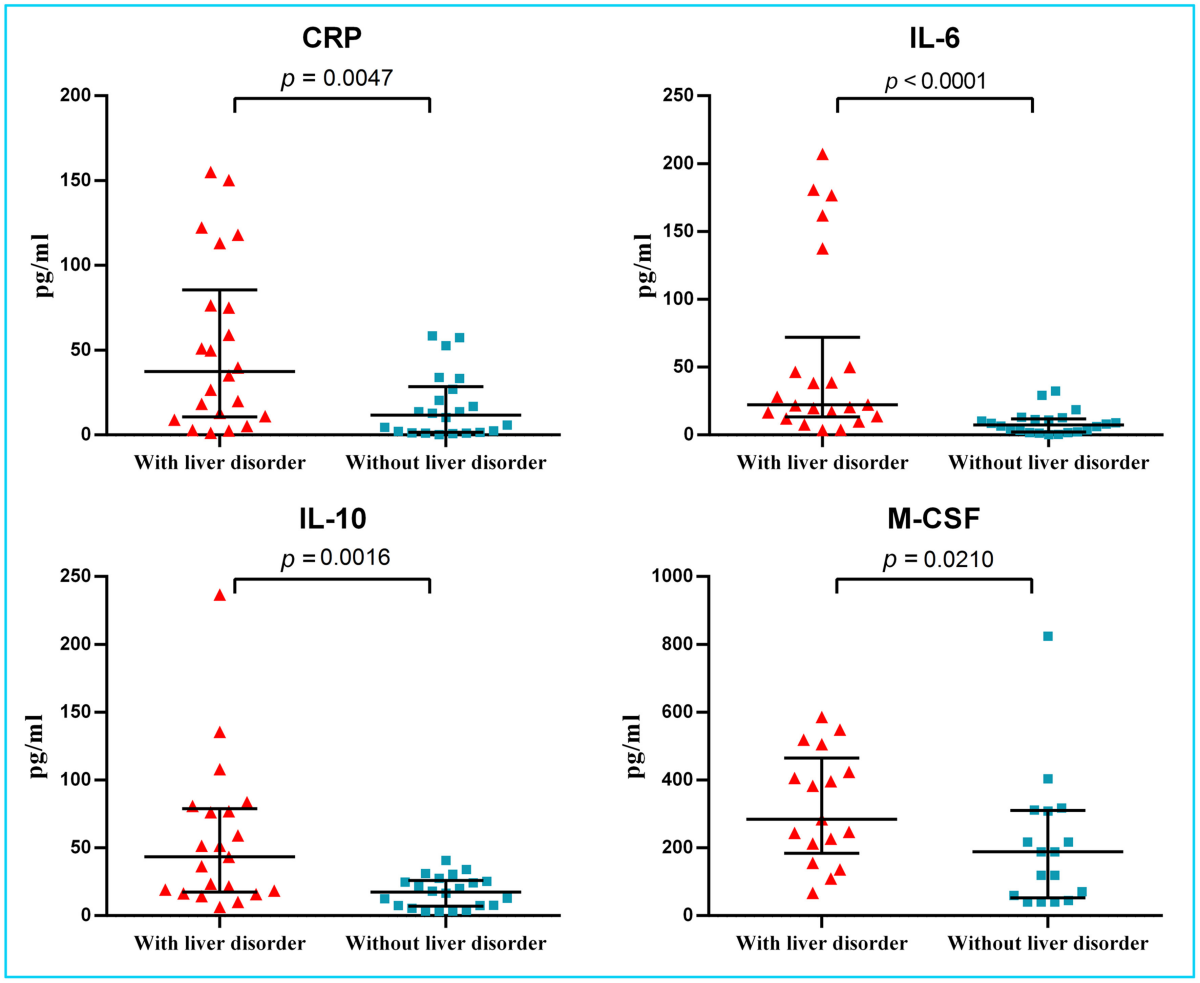
Levels of CRP and several cytokines in plasma of COVID-19 patients with and without liver disorder. Significantly increased CRP levels and higher levels of IL-6, IL-10, and M-CSF were found in COVID-19 patients with liver disorder (*n* = 22) than those without liver disorder (*n* = 22). CRP, C-reactive protein; IL, interleukin; M-CSF, macrophage colony-stimulating factor.

Before hospitalization, four of 22 patients with and two of 22 patients without liver disorders were temporarily treated with a non-steroidal anti-inflammatory drug. The median time from symptom onset to hospitalization was four (IQR 2.3–6.0) days. On admission, the median level of ALT in the liver disorder group was 58.5 U/L, with one value in excess of 200 U/L (264 U/L), but no patient had overt bilirubinemia. No significant differences were seen between the two groups in the onset of symptoms, disease severity, or CT image features (Table 1). Most laboratory tests did not present a distinction between two groups. Lymphopenia was found in 17 (77%) of 22 patients with liver disorder and eight (36%) of 22 patients without liver disorder (*p* = 0.006) (Table 2). The median level of C-reactive protein (CRP) was higher in the hepatic disorder group, with a significantly higher concentration of IL-6, IL-10, and M-CSF (Figure 1). All measured cytokines and chemokines are presented in detail in Figure S1. Excluding three patients with missing plasma samples, viremia was only detected in three (7%) of 41 patients, that is, in two patients with liver disorder and in one without.

**Table 1:** Demographics and baseline characteristics of patients with COVID-19.

**Characteristics**	**Total *N* = 44**	**With liver disorder *N* = 22**	**Without liver disorder *N* = 22**	***P*-value**
Age, years	53.0 (39.0, 64.5)	55.0 (46.8, 69.3)	50.5 (31.0, 63.5)	0.20
≤ 60	29 (66)	16 (73)	13 (59)	0.34
>60	15 (34)	6 (27)	9 (41)	
Sex				0.75
Men	15 (34)	8 (36)	7 (32)	
Women	29 (66)	14 (64)	15 (68)	
**Exposure history**
Exposure to Hubei	13 (30)	9 (41)	4 (18)	0.099
Exposure to confirmed COVID-19 patients	31 (71)	15 (68)	16 (73)	0.741
Cluster or family cases	38 (86)	18 (82)	20 (91)	0.66
Estimated incubation period				0.297
1–4 days	16 (36)	6 (27)	10 (46)	
≥5 days	19 (43)	12 (55)	7 (32)	
Unsure	9 (21)	4 (18)	5 (23)	
**Previous medical history**
HBV co-infection	1 (2)	1 (5)	0	1.00
Non-alcoholic fatty liver disease	2 (5)	1 (5)	1 (5)	1.00
Alcohol abuse	2 (5)	1 (5)	1 (5)	1.00
Digestive system disease	2 (5)	1 (5)	1 (5)	1.00
Hypertension	10 (23)	7 (32)	3 (14)	0.15
Cardiovascular disease	3 (7)	2 (9)	1 (5)	1.00
Diabetes	5 (11)	3 (14)	2 (9)	1.00
Chronic pulmonary disease	0			
Malignancy	3 (7)	2 (9)	1 (5)	1.00
History of surgery	12 (27)	8 (36)	4 (18)	0.176
History of allergy	6 (14)	3 (14)	3 (14)	1.00
Use of non-steroidal anti-inflammatory drug before admission	6 (14)	4 (18)	2 (9)	0.66
Clinical typing				1.00
Mild or moderate	32 (73)	16 (73)	16 (73)	
Severe or critical	12 (27)	6 (27)	6 (27)	
Days from illness onset to admission	4.0 (2.3, 6.0)	3.0 (2.0, 5.0)	5.0 (2.8, 7.0)	0.173
0–3	21 (48)	13 (59)	8 (36)	0.271
4–5	11 (25)	5 (23)	6 (27)	
>5	12 (27)	4 (18)	8 (36)	
**Symptoms and signs**
Fever	37 (84)	18 (82)	19 (86)	1.00
Highest temperature, °C	38.5 (37.8, 38.9)	38.5 (37.7, 38.8)	38.3 (37.8, 39.0)	0.751
<38.5	21 (48)	9 (41)	12 (55)	0.365
≥38.5	23 (52)	13 (59)	10 (46)	
Cough	34 (77)	16 (73)	18 (82)	0.472
Sticky expectoration	20 (46)	10 (46)	10 (46)	1.00
Hemoptysis	1 (2)	0	1 (5)	1.00
Chill	4 (9)	4 (18)	0	0.116
Headache	2 (5)	2 (9)	0	0.469
Stuffed nose	4 (9)	2 (9)	2 (9)	1.00
Fatigue	14 (32)	9 (41)	5 (23)	0.195
Myalgia and arthralgia	8 (18)	7 (32)	1 (5)	0.051
Loss of appetite	12 (27)	7 (32)	5 (23)	0.498
Nausea	6 (14)	2 (9)	4 (18)	0.66
Diarrhea	1 (2)	1 (5)	0	1.00
Shortness of breath	18 (41)	8 (36)	10 (46)	0.54
Systolic pressure, mmHg	120.0 (112.0, 130.0)	120.5 (108.0, 140.5)	120.0 (112.8, 130.0)	0.75
Respiratory rate >24 breaths/min	1 (2)	1 (5)	0	1.00
Arterial oxygen pressure <60 mmHg	8 (18)	4 (18)	4 (18)	1.00
CT findings				0.41
Normal or unilateral pneumonia	7 (16)	2 (9)	5 (23)	
Bilateral diffused ground-glass opacity	37 (84)	20 (91)	17 (77)	

*Data are medians (IQRs) or total numbers (percentages). P-values that compare patients with and without liver disorder are drawn from the χ^2^ test, Fisher's exact test, or the Mann–Whitney U-test*.

**Table 2:** Laboratory results for patients with COVID-19 on admission to the hospital.

**Characteristics**	**Total *N* = 44**	**With liver disorder *N* = 22**	**Without liver disorder *N* = 22**	***P*-value**
White blood cell count, × 10^9^/L	4.3 (3.5, 5.6)	4.2 (2.9, 6.1)	4.3 (3.5, 5.4)	0.972
≥4	26 (59)	12 (55)	14 (64)	0.54
<4	18 (41)	10 (46)	8 (36)	
Neutrophil count, × 10^9^/L	2.8 (1.9, 4.0)	2.9 (1.8, 4.7)	2.5 (2.0, 3.4)	0.348
Lymphocyte count, × 10^9^/L	0.9 (0.7, 1.5)	0.8 (0.5, 1.0)	1.4 (0.9, 1.8)	0.001
≥1.0	19 (43)	5 (23)	14 (64)	0.006
<1.0	25 (57)	17 (77)	8 (36)	
Monocyte count, × 10^9^/L	0.3 (0.2, 0.4)	0.2 (0.2, 0.3)	0.3 (0.2, 0.4)	0.069
Hemoglobin, g/L	133.0 (123.3, 141.8)	134.5 (124.3, 142.8)	132.5 (122.5, 140.5)	0.474
Platelet count, × 10^9^/L	200.0 (161.0, 239.0)	186.5 (163.0, 220.3)	215.5 (146.8, 252.5)	0.372
Platelet hematocrit, %	0.2 (0.2, 0.2)	0.2 (0.2, 0.2)	0.2 (0.2, 0.3)	0.805
Alanine aminotransferase, U/L	42.0 (23.0, 58.8)	58.5 (46.5, 102.3)	23.0 (16.8, 28.0)	<0.001
≤ 80	38 (86)	16 (73)	22 (100)	0.028
>80	6 (14)	6 (27)	0	
Aspartate aminotransferase, U/L	31.0 (23.0, 46.8)	40.0 (28.8, 55.8)	25.5 (17.8, 34.3)	0.003
Albumin, g/L	37.2 (32.9, 39.3)	36.6 (32.7, 38.7)	37.3 (33.9, 40.5)	0.385
Albumin/globulin	1.0 (0.8, 1.2)	1.0 (0.8, 1.1)	1.1 (0.9, 1.3)	0.139
Total bilirubin, μmol/L	10.5 (7.4, 13.2)	9.6 (7.2, 13.0)	11.1 (7.4, 13.7)	0.432
Creatinine, μmol/L	62.0 (53.5, 74.0)	64.0 (56.8, 73.3)	56.5 (51.0, 76.0)	0.496
Estimated glomerular filtration rate, mL/min	99.1 (91.5, 110.6)	96.5 (87.2, 109.3)	103.2 (94.8, 113.6)	0.25
≥90	34 (77)	16 (73)	18 (82)	0.472
<90	10 (23)	6 (27)	4 (18)	
Potassium, mmol/L	3.6 (3.4, 4.0)	3.7 (3.4, 4.1)	3.6 (3.5, 3.9)	0.907
Sodium, mmol/L	137.1 (135.0, 139.3)	136.7 (132.5, 138.3)	137.8 (136.2, 140.2)	0.033
Chloride, mmol/L	101.4 (99.7, 103.4)	100.3 (99.2, 101.9)	103.0 (101.1, 104.6)	0.002
Coagulation profile				
Prothrombin activity, %	75.0 (70.0, 82.0)	76.0 (73.3, 81.3)	75.0 (69.5, 84.0)	0.972
≥80	15 (34)	7 (32)	8 (36)	0.75
<80	29 (66)	15 (68)	14 (64)	
Prothrombin time, sec	12.6 (11.9, 13.2)	12.5 (12.0, 12.9)	12.6 (11.7, 13.4)	0.981
>12.8	13 (30)	5 (23)	8 (36)	0.322
≤ 12.8	31 (71)	17 (77)	14 (64)	
Activated partial thromboplastin time, sec	33.0 (29.0, 35.4)	33.3 (30.3, 35.3)	32.9 (28.7, 35.9)	0.742
>36.5	2 (5)	0	2 (9)	0.469
≤ 36.5	42 (96)	22 (100)	20 (91)	
CRP, mg/L	17.6 (3.3, 52.3)	37.4 (10.6, 85.6)	11.6 (1.5, 28.5)	0.005
≥30	17 (39)	12 (55)	5 (23)	0.03
<30	27 (61)	10 (46)	17 (77)	
Procalcitonin, ng/mL	0.12 (0.1, 0.13)	0.13 (0.11, 0.15)	0.11 (0.1, 0.12)	0.003
Creatine kinase, U/L	83.5 (46.0, 133.5)	98.0 (51.0, 191.5)	66.0 (45.8, 119.8)	0.24
Troponin-I, ng/mL	0.01 (0.01, 0.02)	0.01 (0.01, 0.02)	0.02 (0.01, 0.02)	0.334

*Data are medians (IQRs) or total numbers (percentages). P-values that compare patients with and without liver disorder are from the χ^2^ test, Fisher's exact test, or the Mann–Whitney U-test*.

## Discussion

Although our study did not reveal any dynamic change or prognosis during the clinical course, as a cross-sectional study, we found that there is no relationship between liver condition and hospital medication.

Previous investigations have indicated that the incidence of viremia in COVID-19 patients ranges from 8 to 15% (8, 9). Our study also found viremia to be uncommon in patients with and those without liver disorder on admission. If viremia is required for SARS-CoV-2 to cause direct liver injury via the ACE2 receptor, SARS-CoV-2 hepatitis could not account for the commonly observed liver disorders in COVID-19 patients. A recent review of seven studies suggested that elevated ALT and AST may not necessarily be hepatogenic (10), as similar liver abnormalities are also thought to be common in other respiratory viral infections. Despite the large number of COVID-19 patients with liver injury, there have been no reports of liver failure caused by this disease (11). From the point of pathology, SARS-CoV-2 RNA has been detected in liver tissue from some fatal cases (12), but other autopsies of COVID-19 patients have not revealed the typical pathological manifestation of viral hepatitis caused by SARS-CoV-2 (13, 14).

However, our findings support the possibility that a dysregulated immune response may be a crucial pathogenic factor in liver disorders. On admission, the levels of CRP and the production of the cytokines IL-6, IL-10, and M-CSF were significantly elevated in patients with a liver disorder. The early study suggested that, with ensuing local inflammation of lung affected areas, secretion of the pro-inflammatory cytokines and chemokines was increasing, such as IL-6, IFNγ, M-CSF, and MCP1, and these would release into the blood of COVID-19 patients (9). In severe patients, levels of IL-2, IL-7, IL-10, granulocyte colony-stimulating factor (G-CSF), and IP-10 elevated more significantly than in mild or moderate patients. A markedly higher levels of IL-6 and IL-10 related with a progression of disease severity and poor prognosis (15). Unlike previous reports (16, 17), we didn't identify the difference of IL-1β, an important cytokine released during pyroptosis, probably due to the early stage of a cross-sectional study. Notably, such derailed non-specific cytokine secretion could easily result in liver disorders, which is common in other infectious disease (18).

In our study, overt bilirubinemia was absent in COVID-19 patients with elevated ALT levels. The overproduction of these inflammation-related cytokines with mild liver function abnormality do not seem to be a typical pattern in known viral, alcohol-, or drug-induced hepatitis. For other coexisting conditions that could influence liver, the four patients in the liver disorder group used non-steroidal anti-inflammatory drug (NSAID) and two patients without liver disorder used NSAID; other patients' liver function did not have a problem with drug interference at admission. The patients with respiratory distress or arterial oxygen pressure <60 mmHg were evenly distributed in each group. There were no patients with multiple organ dysfunction at admission. Thus, the commonly observed liver disorders cannot be attributed to the above causes. Taken together, these indications lead to the conclusion that the observed liver disorder is more likely a secondary injury of an aberrant immune response, as has been shown for other human coronavirus infections (19).

Our study had several limitations. First, the viremia was checked qualitatively, not quantitatively, but due to its rarity, a qualitative test is not unacceptable for viremia detection. Second, we did not test GGT or ALP, so most patients had no ultrasonic records if they had not declared a history of liver diseases. After obtaining a medical history through inquiries, it appeared possible that fatty liver, chronic cholecystitis, or even compensatory cirrhosis might have been present in some cases but was not diagnosed. Third, except for the remarkable change in pro-inflammatory cytokines, functional exhaustion and reduced diversity of T cells is another immune dysfunction profile in COVID-19 patients, but has not been included in the current study (20). Finally, we recognize the small size of the sample; the available data are insufficient to distinguish enough types of cytokines' differences between two groups. We nevertheless suggested that our clinical design might be useful for investigating whether SARS-CoV-2 directly targets other specific extrapulmonary organs (21).

In conclusion, our study provides evidence that liver disorders in COVID-19 patients results from a systemic immune response, but there may not be direct virus-induced hepatic cell damage. It should be kept in mind that liver disorders, even during the early course of COVID-19, indicate the occurrence of a drastic cytokine release status, which may be a sign of the possible fierce inflammation condition and rapid disease progression.

## Data Availability Statement

The datasets presented in this study can be found in online repositories. The names of the repository/repositories and accession number(s) can be found in the article/ Supplementary Material.

## Ethics Statement

The studies involving human participants were reviewed and approved by Beijing Youan Hospital, Capital Medical University Ethical Committee. Written informed consent to participate in this study was provided by the participants' legal guardian/next of kin. Written informed consent was obtained from the individual(s), and minor(s)' legal guardian/next of kin, for the publication of any potentially identifiable images or data included in this article.

## Author Contributions

YG, QL, YF, YC, HW, RJ, and XH contributed to the study design, patient recruitment, data collection, data statistical analysis, data interpretation, literature search, and writing of the manuscript. XH, TZ, and LL contributed to the accuracy of the data analyses. HS and DC contributed to the laboratory tests and analyses. All authors reviewed and approved the final version of the manuscript.

## Conflict of Interest

The authors declare that the research was conducted in the absence of any commercial or financial relationships that could be construed as a potential conflict of interest.
