# Supplementary material for: Preliminary Exploration of the Cause of Liver Disorders During Early Stages in COVID-19 Patients
Source: Front Med (Lausanne). 2020 Aug 7;7:501. doi: 10.3389/fmed.2020.00501 (PMC7443567; doi:10.3389/fmed.2020.00501)
Supplement: Supplementary file 1 [file Data_Sheet_1.docx]

**Supplementary Appendix**


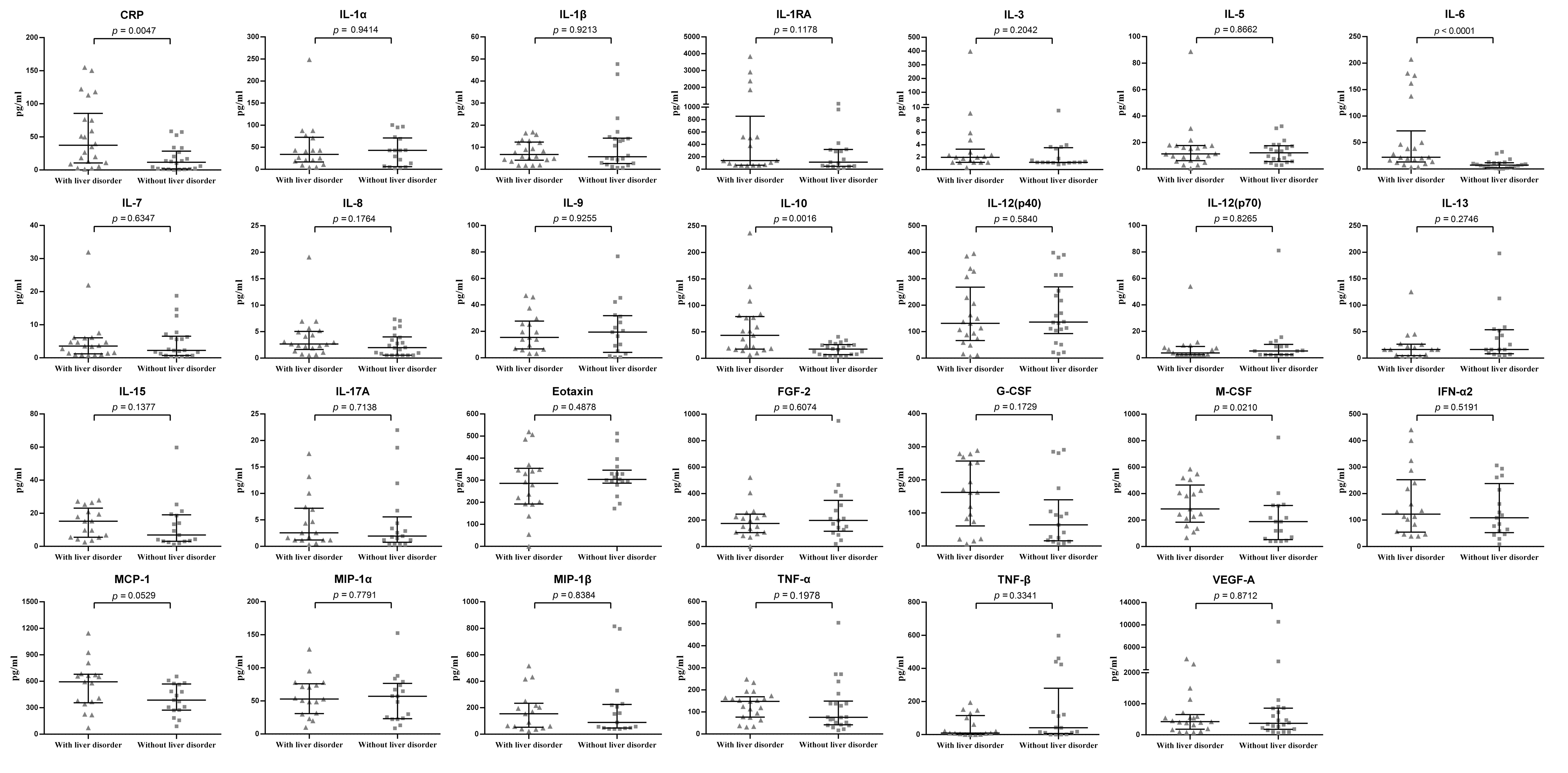


**Figure S1. Serum levels of C-reactive protein, cytokines, and chemokines among patients with and without liver disorder**

The vertical axes differ for each of the factors to accommodate the extreme variation in the normally distributed range of concentrations. The data are presented as medians and interquartile ranges. CRP = C-reactive protein; IL = interleukin; FGF = fibroblast growth factors; G-CSF = granulocyte colony-stimulating factor; M-CSF = macrophage colony-stimulating factor; IFN-γ = interferon γ; MCP = monocyte chemoattractant protein; MIP = macrophage inflammatory protein; TNF-α = tumor necrosis factor α; VEGF = vascular endothelial growth factor. The liver disorder group showed a prominent increase in CRP level and higher concentrations of IL-6, IL-10, and M-CSF among the 26 cytokines and chemokines.
